# Measuring long-term disease control in patients with atopic dermatitis: A validation study of well-controlled weeks

**DOI:** 10.1016/j.jaci.2017.02.043

**Published:** 2017-12

**Authors:** Sinéad M. Langan, Beth Stuart, Lucy Bradshaw, Jochen Schmitt, Hywel C. Williams, Kim S. Thomas

**Affiliations:** a Faculty of Epidemiology and Population Health, London School of Hygiene and Tropical Medicine, London, United Kingdom; b Faculty of Medicine, University of Southampton, Southampton, United Kingdom; c Nottingham Clinical Trials Unit, University of Nottingham, Nottingham, United Kingdom; d Centre of Evidence Based Dermatology, University of Nottingham, Nottingham, United Kingdom; e Centre for Evidence-based Healthcare, Medical Faculty Carl Gustav Carus, TU Dresden, Dresden, Germany

**Keywords:** Atopic dermatitis, long-term control, outcome measures, AD, Atopic dermatitis, CLOTHES, Clothing for the Relief of Eczema Symptoms, EASI, Eczema Area and Severity Index, HOME, Harmonising Outcome Measures in Eczema, POEM, Patient Orientated Eczema Measure, SASSAD, Six Signs, Six Areas Atopic Dermatitis Scale, SWET, Softened Water Eczema Trial, TIS, Three Item Severity, UK, United Kingdom, WCW, Well-controlled week

## Abstract

**Background:**

Because atopic dermatitis (AD) is a relapsing remitting disease, assessing long-term control is important. Well-controlled weeks (WCWs) have been used to assess asthma long-term control but have never been validated for AD.

**Objectives:**

We sought to assess the feasibility, validity, and interpretability of WCWs in patients with AD.

**Methods:**

Three studies of patients with moderate-to-severe AD, including 4 to 6 months of daily/weekly symptom and treatment use data, were evaluated (study A, n = 336; study B, n = 60; and study C, n = 224). WCWs were defined by worsening symptoms and increased medication use. Feasibility, construct validity, and interpretability of WCWs were determined by assessing missing data, association with validated AD outcomes, and floor and ceiling effects. Analysis used linear and logistic regression.

**Results:**

WCWs were feasible to collect: 95.2% (study A) and 94.7% (study B) contributed data for at least half of the weekly data points, and 93.2% and 88.7% contributed to all data points up to 4 months. WCWs were significantly associated with validated AD severity instruments, including patient-orientated outcome measures and objective signs (Eczema Area and Severity Index, Three Item Severity Score, and Six Signs, Six Areas Atopic Dermatitis Scale). The odds of experiencing a WCW if AD severity was clear/mild was 5.8 (95% CI, 3.5-9.7), 1.9 (95% CI, 0.8-4.4), and 8.1 (95% CI, 4.5-14.6) in studies A, B, and C, respectively. WCWs were associated with ceiling effects: 31.6% (study A) and 37.5% (study B) of participants had no WCWs more than 90% of the time.

**Conclusions:**

WCWs are valid and feasible for measuring long-term control in AD trials. However, ceiling effects and burden of data collection can limit use.

Atopic dermatitis (AD; also known as atopic eczema or eczema) is the most common inflammatory disease of childhood, affecting 20% of children at some point in their lives and approximately 3% of adults.^1^, ^2^ It is characterized by a chronic relapsing remitting disease course. Flares are a major component of disease morbidity, with major effects on patients and their families.^3^ Capturing chronicity of disease and measures of longer-term disease control is an important clinical outcome and is becoming increasingly important with the drive for more pragmatic, longer-term comparative effectiveness trials.^4^

Research in AD has been hampered by the use of a vast array of outcome measures, the majority of which have not been adequately validated.^5^ The Harmonising Outcome Measures in Eczema (HOME) initiative (www.homeforeczema.org) is an international collaborative effort comprising international stakeholders, who are working together to establish consensus over a core outcome set for AD research. Measuring long-term control has been identified as a core outcome domain for clinical trials in AD, but at present, there is no established and validated measure to do this.^4^, ^6^

To address the lack of an accepted and validated way of measuring long-term control in AD, our group previously proposed a definition for well-controlled weeks (WCWs) based on the literature in the field of asthma.^7^ The proposed definition for a WCW is based on having 2 days or fewer with (1) symptoms greater than a prespecified level and (2) escalation of treatment required. Hence WCWs reflect a behavioral response to the worsening of AD. WCWs are distinct from totally controlled weeks, where no symptoms are observed during a week. Thus a WCW is based on the concept that if the chronic disease is only associated with increased symptoms for 2 or fewer days that week, it is relatively well controlled. Hence, if a study participant has fewer WCWs, they have worse disease control, whereas those with many WCWs have well-controlled disease. This definition of WCWs has not previously been validated or evaluated in an AD research setting.^7^, ^8^ There is little clarity on how WCWs should be measured or interpreted and how they relate to other validated outcome measures for AD.

This article reports our experiences of using WCWs in 3 clinical studies (2 randomized controlled trials and 1 observational study): the Softened Water Eczema Trial (SWET), an observational study of environmental triggers of disease flares in childhood AD, and the Clothing for the Relief of Eczema Symptoms (CLOTHES) trial.^9^, ^10^, ^11^ Study objectives were as follows: (1) to assess the feasibility of WCWs as a measure of long-term AD control; (2) to explore the association between WCWs and other validated AD outcome severity instruments (patient-reported severity and objective severity scales); and (3) to evaluate the interpretability of WCWs by examining the floor and ceiling effects and the relationship between WCWs and eczema severity.

Floor and ceiling effects occur when a high proportion of study participants experience the best or worst outcome for the majority of the study period, respectively. In this study floor effects occurred if a substantial proportion experienced a state of WCWs for the majority of the study period. Conversely, ceiling effects occurred when a substantial proportion of patients did not achieve a WCW for the majority of the study period. Both floor and ceiling effects are problematic because they hamper the ability to distinguish at extremes of disease severity.

## Methods

Ethics approval was not required for this study because it represents a secondary analysis of existing data sets from previously conducted and ethically approved studies.

### Data sources

Data from 3 United Kingdom (UK)–based studies (2 funded by the National Institute for Health Research and 1 funded by the BUPA Foundation) have been used to inform these analyses. The data sets include children with moderate-to-severe AD who were recruited in both primary and secondary care settings.

#### Study A: SWET^12^

The SWET trial was a randomized controlled trial of 4 months' duration involving 336 children with moderate-to-severe AD aged between 6 months and 16 years recruited between 2007 and 2009. Children were recruited from 8 UK secondary care centers. Participants received normal care plus an ion-exchange water softener or normal care alone. Participants had clinic visits at baseline and 4, 12, and 16 weeks. Data to define WCWs were collected daily by using paper diaries. Validated AD severity scales (the Patient-Orientated Outcome Measure [POEM], Six Signs, Six Areas Atopic Dermatitis [SASSAD] scale, and Three Item Severity [TIS] score) were completed during the clinic visits.^13^, ^14^, ^15^

#### Study B: observational study to identify flare triggers^9^

This study was a 6-month prospective cohort study involving 60 children with moderate-to-severe AD assessing associations between environmental exposures and disease flares in patients with AD between 2006 and 2007. Participants were aged up to 15 years and recruited from a single UK center. Participants had clinic visits at baseline and monthly for 6 months. Data to define WCWs were collected by using daily electronic diaries. Validated AD severity scales (POEM and TIS) were completed during clinic visits.

#### Study C^11^

The CLOTHES trial was a randomized controlled trial of 6 months' duration involving 300 children with moderate-to-severe AD aged 1 to 15 years and recruited from 5 secondary care centers in the UK between 2013 and 2015. Participants received standard care plus silk therapeutic clothing or standard care alone. Participants had clinic visits at baseline and 8, 16, and 24 weeks and completed weekly online questionnaires. WCWs were not a specified outcome for the CLOTHES trial; however, data necessary to define WCWs were available from weekly online questionnaires and clinic visits, making inclusion in this validation study possible. Validated AD severity scales (POEM, the Eczema Area and Severity Index [EASI], and TIS) were completed during the clinic visits.

### Defining WCWs

We previously suggested that a WCW should be defined where treatment escalation (stepping up of treatment) was used for only 2 or fewer days for that week and where symptoms were increased to greater than a prespecified level for 2 or fewer days during that week.^8^ Valid symptom assessment tools could include either a patient global assessment or a self-reported bother/itch/scratch score. The prespecified symptom level was proposed as being greater than 1 on a 5-point Likert scale (0-4) or greater than 4 on an 11-point visual analog scale (0-10).

We defined escalation of treatment as any additional treatment that had been specified in the study protocol to deal with disease deterioration. In some study designs, study treatment is used as an “as-required” treatment in response to disease worsening, and therefore study treatment could be considered treatment escalation. If a treatment was used for less than 2 days per week as proactive therapy for the prevention of flares, this was not considered escalation of treatment.^16^ In those using low-potency steroids, escalation could include increasing the steroid potency to moderate or potent topical steroids. In those using potent steroids, stepping up to superpotent topical steroids or using wet wraps could constitute an escalation.

Table I^9^, ^11^, ^13^, ^14^, ^15^, ^17^, ^18^ provides a summary of how WCWs were defined in each of the included studies. For studies A and B, escalation of treatment was defined on an individual basis for each child by parents in conjunction with study investigators at the start of the study. For study C, the number of days of topical corticosteroids each week was used to define treatment escalation.

**Table I:** Data available from included studies

Data captured	Study A^17^	Study B^9^	Study C^11^
How was moderate-to-severe eczema defined?	SASSAD score ≥10	≥3 Flare-ups in previous 6 mo	Nottingham Eczema Severity Scale (NESS) score of ≥9
Definition of WCW used∗	WCW: ≤2 d where stepping up of treatment was required and ≤2 d with a bother score >4	WCW: ≤2 d where stepping up of treatment was required and ≤2 d with a bother score >4	WCW: ≤2 d when topical corticosteroids used and global bother score ≤4 for the week
Bother score (0-10): “How much bother did your (your child's) eczema cause today?”	Daily in paper diaries	Daily in electronic diaries	Bother over the last week assessed at baseline and weeks 8, 16, and 24
Scratch score (0-10): “How much did you (your child) scratch today?”	Daily	Daily	—
Treatment “stepped up” (individually defined at start of study [yes/no])	Daily	Daily	—
Topical corticosteroid use (yes/no)	Daily (yes/no)	—	Weekly (no. of days in last week)
Topical calcineurin inhibitor use (yes/no)	Daily	—	Weekly (no. of days in last week)
POEM scores^13^ (range, 0-28)†	Baseline and weeks 4, 12, and 16	Baseline and weeks 4, 8, 12, 16, 20, and 24	Weekly
TIS scores^15^ (range, 0-9)†	Weeks 12 and 16	Baseline and weeks 4, 8, 12, 16, 20, and 24	Baseline and weeks 8, 16, and 24
SASSAD scores^14^ (range, 0-108)†	Weeks 12 and 16	—	—
EASI scores^18^ (range, 0-72)†	—	—	Baseline and weeks 8, 16, and 24

∗ Higher WCWs represents better disease control.

† Higher scores denote more severe disease.

WCW data were collected daily for studies A and B and weekly for study C. For study C, data on the number of days that topical corticosteroids were used were collected weekly, and global bother over the last week was collected every 2 months. As such, WCWs in study C could only be calculated at 8, 16, and 24 weeks, despite the availability of weekly treatment use data.

Details of other outcomes related to eczema severity collected in the included studies are outlined in Table I.

### Evaluation of WCWs and hypotheses tested

#### Feasibility of collecting WCWs in clinical studies

• Assessments were based on the amount of missing data for each of the included data sets.
• WCWs were judged to be feasible to collect if more than 50% of participants completed at least half of the daily/weekly questionnaires and if more than 80% of participants were eligible for inclusion in the repeated-measures analysis of WCWs (studies A and B only).

#### Association between WCWs and other commonly used AD outcome scales (construct validity)

• The degree to which WCWs relate to other validated outcome scales (POEM, EASI, TIS, and SASSAD) was determined.
• We hypothesized that participants reporting a WCW would have lower severity scores for AD symptoms (POEM) and AD signs (EASI, TIS, and SASSAD) for that week.

#### Interpretability of WCWs

• Assessment was done by examining the distribution of WCWs to look for floor and ceiling effect and by assessing the odds of experiencing a WCW according to eczema severity (using previously validated POEM bandings for mild, moderate, and severe disease).^19^
• WCWs were assumed to have problematic ceiling effects if more than 15% of participants experienced no WCWs more than 90% of the time or floor effects if more than 15% of participants experienced a WCW more than 90% of the time.^20^

### Statistical methods

#### Data management

The 3 data sets were analyzed individually to explore the consistency and replication of our findings across different data sets. Analysis of data set A was considered exploratory, and analyses of data sets B and C were considered confirmatory.

For study C, we included participants who had completed weekly questionnaires (providing data on topical corticosteroid use and POEM scores) up to 3 days before a clinic visit or 1 day after the clinic visit to ensure that data were reported in the same time period as the disease severity measures (EASI and TIS) and bother scores, which were captured during the 2-month clinic visits. This meant that 224 (75%) of the 300 trial participants contributed to this validation study. As a result, study C was excluded from the analysis of missing data (because all had available data to be included in the study) and floor and ceiling effects (because only 3 data points were available).

#### Feasibility: missing data

The quantity of missing data was determined for WCWs in studies A and B. The following rules were developed to handle missing data:

• If there were 3 days or more with either a bother score of greater than 4 or where “stepping up” was required, then the week was not defined as a WCW.
• If only 1 day had a bother score of greater than 4 and there is only 1 missing day, then the week was classed as a WCW; the same rules apply for treatment escalation (stepping up of treatment).

#### Construct validity: association between WCWs and validated scales

The strength and direction of association between WCWs and other measures of disease severity (POEM, TIS, SASSAD, and EASI scores) was assessed for weeks 4, 12, and 16 in study A; weeks 4, 8, 12, 16, 20, and 24 in study B; and weeks 8, 16, and 24 in study C.

Because data were captured at different time points in the 3 studies (Table I), the primary analysis included participants with data for at least 2 of the time points.

Given the repeated-measures nature of the study, data were analyzed by using a mixed linear models in Stata software (version 14; StataCorp, College Station. Tex). This allows participants who have missing data to contribute information for any periods for which they have data at the same time point for both WCWs and the validated severity instrument; no assumptions were made about missing values.

#### Interpretability

The proportion of the study period spent with a WCW was calculated for all participants who contributed data for at least 50% of the study period to explore whether WCWs were subject to floor and ceiling effects (studies A and B only).

Predefined categorical bands for POEM scores were used to evaluate clinical interpretability: clear/mild (0-7), moderate (8-16), and severe/very severe (17-28) AD.^19^ Participants needed to have data on WCWs and POEM scores for at least 1 time point after baseline to contribute to the analysis. The relationship between POEM severity and WCWs was determined by using mixed logistic regression, with the moderate severity group as the reference group.

#### Power

No formal sample size estimation was conducted because the sample size for this study was pragmatic based on data availability. A sample size of greater than 100 participants per analysis has been recommended as sufficient for validation studies.^21^

## Results

Overall, 608 participants contributed to the analyses (study A, n = 325; study B, n = 59; and study C, n = 224). Baseline characteristics of included participants are summarized and demonstrate similar baseline characteristics, although study B is significantly smaller than studies A and C (Table II).

**Table II:** Baseline characteristics of the subjects included in each study

	Study A	Study B	Study C
No. of participants	325	59∗	224
Age, no. (%)			
Mean age (y [SD])	5.40 (4.11)	7.3 (4.8)	5.1 (3.6)
Less than 3 y	94 (29)	14 (23)	73 (33)
3-6 y	118 (36)	13 (22)	78 (35)
≥7 y	113 (35)	33 (55)	73 (33)
Sex, no. (%)			
Male	185 (57)	32 (53)	128 (57)
Female	140 (43)	28 (47)	96 (43)
Ethnicity, no. (%)			
White	253 (78)	38 (63)	183 (82)
Asian	29 (9)	14 (23)	10 (4)
Black	10 (3)	4 (7)	4 (2)
Mixed	19 (6)	4 (7)	18 (8)
Other	12 (4)	0	9 (4)
Not stated/unknown	2 (1)	0	0
AD severity (POEM)^13^			
Mean (SD)	16.7 (5.81)	12.4 (6.4)	17.0 (5.2)
AD severity: Clinical signs			
SASSAD score, no. (%)^14^			
Mean (SD)	25.5 (13.40)	NA	NA
10-19	138 (43)	NA	NA
>20	186 (57)	NA	NA
EASI score^18^			
Mean (SD)	NA	NA	10.5 (9.2)
TIS score^15^			
Mean (SD)	3.9 (1.8)	3.1 (1.5)	4.9 (1.8)

*NA*, Not applicable.

∗ One participant was unable to be included because he or she contributed no data.

### Objective 1: Feasibility of WCWs as a measure of long-term control

Testing the hypothesis that more than 50% of participants would complete at least half of the daily/weekly questionnaires during the study period, we found high completion rates for WCWs. In study A 320 (95.2%) of 336 participants contributed WCW data for more than half of the 16-week study period, and 325 (97%) of 336 had at least 1 WCW after baseline. In study B 56 (94.7%) of 59 contributed WCW data for more than half of the 24-week study period. In study A sufficient data were available to calculate a WCW 94.5% of the time at 3 months and 93.2% of the time at 4 months. For study B, the data were available 91.9% of the time at 3 months and 88.7% of the time at 4 months.

Testing the hypothesis that at least 80% of participants would be eligible for inclusion in a repeated-measures analysis (assuming that participants could be included if they contributed at least 1 data point for WCWs after baseline), most participants were able to be included (97% in study A and 100% in study B).

### Objective 2: Association between WCWs and other commonly used AD outcome scales

The hypothesis that participants reporting a WCW would have lower AD severity scores for the corresponding week was supported. For all 3 studies, POEM and TIS scores were lower in subjects with a WCW compared with those who did not have a WCW (*P* < .05 for study B and *P* < .01 for studies A and B, Table III). In studies A and C, in which data for SASSAD and EASI scores were available, a similar pattern was observed (Table III).

**Table III:** Strength and direction of the association between WCWs and other AD outcome measures (construct validity)

	Study A (n = 325)			Study B (n = 59)			Study C (n = 224)		
	Mean (SD) score for those with a WCW	Mean (SD) score in those without a WCW	Difference in score for those with a WCW compared with those without (95% CI)	Mean (SD) score for those with a WCW	Mean (SD) score in those without a WCW	Difference in score for those with a WCW compared with those without (95% CI)	Mean (SD) score for those with a WCW	Mean (SD) score in those without a WCW	Difference in score for those with a WCW compared with those without (95% CI)
POEM score (range, 0-28)∗	9.20 (5.75)	14.68 (6.37)	−4.28 (−5.08 to −3.48)	9.17 (5.92)	13.82 (6.49)	−1.92 (−3.26 to −0.58)	6.83 (5.34)	14.25 (6.29)	−5.98 (−7.36 to −4.59)
TIS score (range, 0-9)∗	2.24 (1.61)	2.98 (1.89)	−0.49 (−0.72 to −0.27)	2.15 (1.48)	3.04 (1.97)	−0.40 (−0.76 to −0.03)	2.83 (1.55)	4.23 (2.00)	−1.20 (−1.66 to −0.74)
SASSAD score (range, 0-108)∗	15.42 (9.98)	23.65 (13.83)	−4.34 (−5.61 to −3.07)		NA	NA		NA	NA
EASI score (range, 0-72)∗		NA	NA		NA	NA	3.51 (5.13)	9.19 (10.19)	−3.24 (−5.16 to −1.31)

*NA*, Not applicable.

∗ High scores denote more severe disease.

### Objective 3: Interpretability of WCWs by examining the floor and ceiling effect and the relationship between WCWs and eczema severity

**Fig 1:**
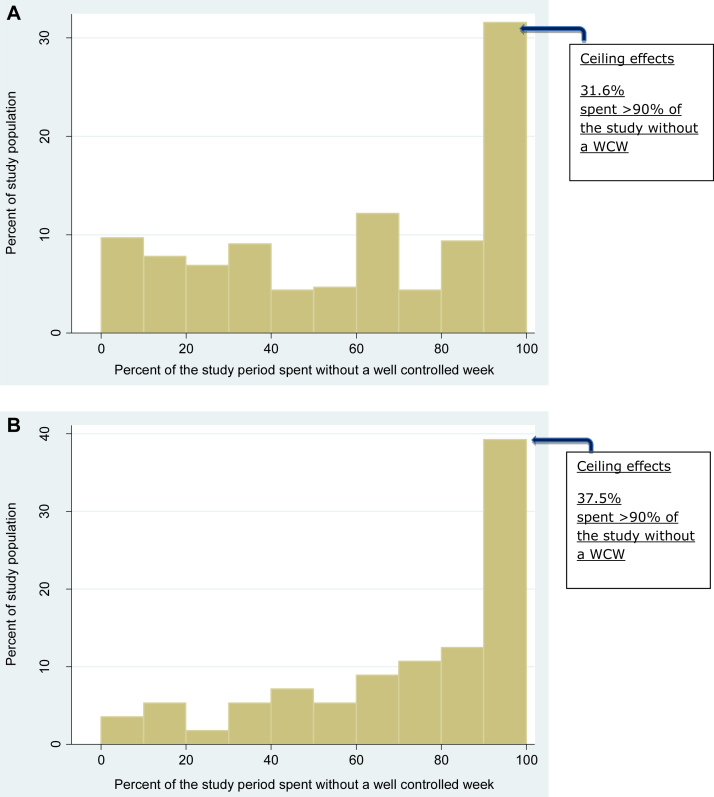
Proportion of the study period spent without a WCW to determine whether there are relevant floor or ceiling effects: **A,** study A; **B,** study B.

The proportion of time spent with a WCW during the study period is shown (Fig 1) and suggests potentially problematic ceiling effects in that more than 15% of the participants spent more than 90% of the time without a WCW (Fig 1); hence a substantial proportion of subjects had poor control of their eczema throughout the study period.

In study A 32 (10%) participants spent more than 90% of the study with a WCW, and 101 (31.6%) spent more than 90% of the study period without a WCW. In study B 2 (3.4%) participants spent more than 90% of the study period with a WCW, and 21 participants (37.5%) spent more than 90% of the study period without a WCW.

The association between WCW scores and AD severity (based on validated POEM bandings) suggests that WCWs are a useful reflection of AD severity. Compared with those with moderate POEM scores, patients with mild or clear AD were more likely to have had a WCW, whereas those with severe or very severe AD were much less likely to have had a WCW (Table IV). For studies A and C these differences were statistically significant, whereas for study B, the relationship was in the same direction but not statistically significant, although this might reflect the smaller sample size of this study.

**Table IV:** Relationship between WCWs and AD severity based on POEM scores as a categorical variable (clear/mild and severe/very severe categories collapsed together)

Categories of POEM scores	Study A: odds ratio for a WCW (95% CI)	Study B: odds ratio for a WCW (95% CI)	Study C: odds ratio for a WCW (95% CI)
Mild (POEM score, 0-7)	5.78 (3.46-9.67)	1.91 (0.82-4.45)	8.08 (4.48-14.59)
Moderate (POEM score, 8-16; reference category)	1.00	1.00	1.00
Severe (POEM score, 17-28)	0.30 (0.17-0.52)	0.57 (0.21-1.52)	0.44 (0.17-1.14)

## Discussion

### Main findings

In this study we have shown that WCWs as defined in the 3 included data sets show good feasibility and construct validity but might be limited by ceiling effects in patients with moderate-to-severe disease. WCWs appear to correlate well with other measures of AD severity, including both POEM and objective outcome instruments capturing AD signs (TIS, EASI, and SASSAD).

Assessment of feasibility is particularly important because measures that are unduly time consuming to collect and analyze or are prone to missing values are unlikely to be recommended for a core outcome instrument.^22^ Measuring long-term control on a daily or weekly basis to define WCWs (by using a combination of symptoms and the need to use AD medications) is a novel approach to determining disease control but is potentially burdensome to both patients and researchers. However, the high completion rates in the included studies would suggest acceptability to patients. It is possible that with increasing use of online tools and mobile phone apps, the technological difficulties of collecting daily or weekly data can be overcome.

Responsiveness is an important criterion for quality assessment of outcome instruments, which has not yet been evaluated. As a binary measure, it might be difficult for a WCW to adequately capture change over time, and the observed ceiling effects could make it difficult to demonstrate meaningful change.^20^ Further work to evaluate responsiveness of WCWs is required.

### Relevance to other studies

It is not yet clear what measure to use and how frequently AD should be assessed to estimate long-term control within the context of a randomized controlled trial, a topic that has been identified as a key priority for future research by a multidisciplinary stakeholder group.^23^ The majority of previous studies have used either patient-reported or objective severity scores assessed 1 to 2 months apart, usually during clinic visits.^5^, ^22^ The optimum frequency of data collection to capture the chronic relapsing nature of AD is not yet known, although it has been reported that assessment of AD severity twice a week provides additional information compared with AD severity collected at 2 months.^24^ The concept of WCWs has been developed specifically to assess the nature of long-term control of eczema. It is a complex measure capturing both the effect of eczema symptoms and the need for treatment escalation. Although capturing the multiple dimensions of eczema control is attractive, using WCWs increases the questionnaire burden on participants and investigators.

Previous work looking at the validation of flare outcomes has suggested that use of topical corticosteroids, calcineurin inhibitors, or both is as sensitive for capturing AD flares as the concept of treatment escalation.^25^ The current study supports this finding because WCWs defined by use of topical corticosteroids/calcineurin inhibitors (in study C) demonstrated similar levels of association with validated scales as those seen in the 2 studies that used escalation of treatment in defining WCWs.

### Strengths and limitations

This study used existing data sets that had been collected originally for another purpose. As a result, some of the analyses were limited by the available data. In the case of study C, the definition of WCWs was *post hoc* and might have influenced the analyses. Nevertheless, we explored the performance of WCWs in the 3 data sets separately and tested predefined hypotheses. We were able to replicate findings in the separate data sets, lending support to the validity of these findings. It is possible that there might have been some overlap of study populations between the included studies because all 3 were recruited in Nottingham, and there was some additional overlap between studies A and C in the recruiting sites. However, the studies were conducted at different periods from 2006 to 2015, and therefore any overlap is likely to be small.

It is currently unclear what proportion of time in a WCW would represent “good control” (which might vary by disease severity), and further work is required to determine whether definitions used to define WCWs can be consistently applied to different studies and populations. Our findings were remarkably consistent across the 3 included studies, but these studies were all conducted in children with moderate-to-severe disease and with participants who were predominantly recruited in secondary care.

### Clinical and research implications

Understanding how to characterize and measure long-term control is a key research priority for the HOME initiative,^6^ and consensus discussions will be taking place at the next HOME meeting in June 2017 (www.homeforeczema.org). WCWs appear to fulfil many of the criteria for consideration as an instrument for measuring long-term control, but this assessment has limitations that require further assessment.

**Key messages**

• WCWs are a composite measure of treatment use and symptoms that have been proposed as a measure of long-term AD control.
• WCWs appear to be closely related to other measures of AD severity, indicating construct validity.
• Capturing data for WCWs can be time consuming, but the limited missing data support acceptability to patients.
• Ceiling effects can be problematic in patients with moderate-to-severe disease and might limit the ability to detect change if participants experience few WCWs during follow-up.

## References

[1] Odhiambo J.A., Williams H.C., Clayton T.O., Robertson C.F., Asher M.I. (2009). ISAAC Phase Three Study Group. Global variations in prevalence of eczema symptoms in children from ISAAC Phase Three. J Allergy Clin Immunol.

[2] Johansson S.G., Bieber T., Dahl R., Friedmann P.S., Lanier B.Q., Lockey R.F. (2004). Revised nomenclature for allergy for global use: Report of the Nomenclature Review Committee of the World Allergy Organization, October 2003. J Allergy Clin Immunol.

[3] Beattie P.E., Lewis-Jones M.S. (2006). An audit of the impact of a consultation with a paediatric dermatology team on quality of life in infants with atopic eczema and their families: further validation of the Infants' Dermatitis Quality of Life Index and Dermatitis Family Impact score. Br J Dermatol.

[4] Schmitt J., Langan S., Stamm T., Williams H.C., panel HOMiEHD (2011). Core outcome domains for controlled trials and clinical recordkeeping in eczema: international multiperspective Delphi consensus process. J Invest Dermatol.

[5] Schmitt J., Langan S., Williams H.C., Network E.D.-E. (2007). What are the best outcome measurements for atopic eczema? A systematic review. J Allergy Clin Immunol.

[6] Chalmers J.R., Simpson E., Apfelbacher C.J., Thomas K.S., von Kobyletzki L., Schmitt J. (2016). Report from the fourth international consensus meeting to harmonize core outcome measures for atopic eczema/dermatitis clinical trials (HOME initiative). Br J Dermatol.

[7] Bateman E.D., Boushey H.A., Bousquet J., Busse W.W., Clark T.J., Pauwels R.A. (2004). Can guideline-defined asthma control be achieved? The Gaining Optimal Asthma ControL study. Am J Respir Crit Care Med.

[8] Langan S.M., Thomas K.S., Williams H.C. (2006). What is meant by a “flare” in atopic dermatitis? A systematic review and proposal. Arch Dermatol.

[9] Langan S.M., Silcocks P., Williams H.C. (2009). What causes flares of eczema in children?. Br J Dermatol.

[10] Thomas K.S., Koller K., Dean T., O'Leary C.J., Sach T.H., Frost A. (2011). A multicentre randomised controlled trial and economic evaluation of ion-exchange water softeners for the treatment of eczema in children: the Softened Water Eczema Trial (SWET). Health Technol Assess.

[11] Harrison E.F., Haines R.H., Cowdell F., Sach T.H., Dean T., Pollock I. (2015). A multi-centre, parallel group superiority trial of silk therapeutic clothing compared to standard care for the management of eczema in children (CLOTHES Trial): study protocol for a randomised controlled trial. Trials.

[12] Thomas K.S., Dean T., O'Leary C., Sach T.H., Koller K., Frost A. (2011). A randomised controlled trial of ion-exchange water softeners for the treatment of eczema in children. PLoS Med.

[13] Charman C.R., Venn A.J., Williams H.C. (2004). The patient-oriented eczema measure: development and initial validation of a new tool for measuring atopic eczema severity from the patients' perspective. Arch Dermatol.

[14] Berth-Jones J. (1996). Six area, six sign atopic dermatitis (SASSAD) severity score: a simple system for monitoring disease activity in atopic dermatitis. Br J Dermatol.

[15] Oranje A.P., Glazenburg E.J., Wolkerstorfer A., De Waard-Van Der Spek F.B. (2007). Practical issues on interpretation of scoring atopic dermatitis: The SCORAD index, objective SCORAD and the three-item severity score. Br J Dermatol.

[16] Schmitt J., von Kobyletzki L., Svensson A., Apfelbacher C. (2011). Efficacy and tolerability of proactive treatment with topical corticosteroids and calcineurin inhibitors for atopic eczema: systematic review and meta-analysis of randomized controlled trials. Br J Dermatol.

[17] Thomas K.S., Sach T.H. (2008). A multicentre randomized controlled trial of ion-exchange water softeners for the treatment of eczema in children: protocol for the Softened Water Eczema Trial (SWET) (ISRCTN: 71423189). Br J Dermatol.

[18] Leshem Y.A., Hajar T., Hanifin J.M., Simpson E.L. (2015). What the Eczema Area and Severity Index score tells us about the severity of atopic dermatitis: an interpretability study. Br J Dermatol.

[19] Charman C.R., Venn A.J., Ravenscroft J.C., Williams H.C. (2013). Translating Patient-Oriented Eczema Measure (POEM) scores into clinical practice by suggesting severity strata derived using anchor-based methods. Br J Dermatol.

[20] De Vet H., Terwee C.B., Mokkink L., Knol D. (2011). Measurement in medicine: a practical guide.

[21] Terwee C.B., Mokkink L.B., Knol D.L., Ostelo R.W., Bouter L.M., de Vet H.C. (2012). Rating the methodological quality in systematic reviews of studies on measurement properties: a scoring system for the COSMIN checklist. Qual Life Res.

[22] Barbarot S., Rogers N.K., Abuabara K., Aubert H., Chalmers J., Flohr C. (2016). Strategies used for measuring long-term control in atopic dermatitis trials: a systematic review. J Am Acad Dermatol.

[23] Schmitt J., Spuls P., Boers M., Thomas K., Chalmers J., Roekevisch E. (2012). Towards global consensus on outcome measures for atopic eczema research: results of the HOME II meeting. Allergy.

[24] Barbarot S., Aubert H., Stadler J.-F. (2016). How patient-reported outcomes can be important in routine practice in children with atopic dermatitis.

[25] Thomas K.S., Stuart B., O'Leary C.J., Schmitt J., Paul C., Williams H.C. (2015). Validation of treatment escalation as a definition of atopic eczema flares. PLoS One.

